# Zingerone alleviates the delayed ventricular repolarization and AV conduction in diabetes: Effect on cardiac fibrosis and inflammation

**DOI:** 10.1371/journal.pone.0189074

**Published:** 2017-12-05

**Authors:** Hany M. El-Bassossy, Wafaa S. Al-Thubiani, Ahmed A. Elberry, Mohammad I. Mujallid, Salah A. Ghareib, Ahmad S. Azhar, Zainy M. Banjar, Malcolm L. Watson

**Affiliations:** 1 Department of Pharmacology and Toxicology, Faculty of Pharmacy, King Abdulaziz University, Jeddah, Saudi Arabia and Faculty of Pharmacy, Zagazig University, Zagazig, Egypt; 2 Department of Biological Sciences, Faculty of Sciences, King Abdulaziz University, Jeddah and Faculty of Applied Sciences, Umm AL-Qura University, Makkah, Saudi Arabia; 3 Department of Pharmacology, Faculty of Medicine, Beni-Suef University, Beni-Suef, Egypt; 4 Department of Biological Sciences, Faculty of Sciences, King Abdulaziz University, Jeddah, Saudi Arabia; 5 Department of Pharmacology, Faculty of Pharmacy, Zagazig University, Zagazig, Egypt; 6 Department of Pediatric, Faculty of Medicine, King Abdulaziz University, Jeddah, Saudi Arabia; 7 Department of Clinical Biochemistry, Faculty of Medicine, King Abdulaziz University, Jeddah, Saudi Arabia; 8 Department of Pharmacy and Pharmacology, University of Bath, Bath, United Kingdom; Max Delbruck Centrum fur Molekulare Medizin Berlin Buch, GERMANY

## Abstract

**Background:**

The study aims to analyse the action of zingerone in diabetes-related cardiac arrhythmias.

**Methods:**

Diabetes was induced by streptozocin while treatment groups received 20 mg/kg zingerone daily. Following extra seven weeks, electrocardiography, extraction of blood, urine and heart for biochemical analysis, histopathology and immunofluorescence were undertaken.

**Results:**

The suppression of QT and QTc prolongation in diabetic rats was indicative of prolonged cardiac repolarisation that was greatly reduced by zingerone treatment. In addition, the reduction in PR interval attested that zingerone improved AV delay in diabetic rats. The fibrogenic transforming growth factor β1 upregulation in diabetic hearts was suppressed by zingerone. The marked glycogen deposition and muscle degeneration seen in diabetic heart sections were also alleviated by zingerone. Furthermore, zingerone prevented the decrease in of the serum anti-inflammatory cytokine adiponectin in diabetics. The heightened levels of oxidative stress markers 8-isoprostane and uric acid in diabetic rats were suppressed. In the diabetic heart, the reduced catalase activity was improved and the excessive expression of angiotensin receptor 1 was inhibited by zingerone.

**Conclusion:**

Cardiac delayed repolarisation and AV conduction in rats with diabetes were halted by zingerone. It appears that inhibition of cardiac fibrosis and associated inflammation-oxidative stress signalling underpins the zingerone effect.

## Introduction

A lifelong metabolic disease accompanied by oxidative stress and hyperglycaemia, diabetes mellitus gives rise to degenerative complications and tissue deterioration in different organs, including the heart [1]. Indeed, diabetes mellitus often proves fatal as a result of cardiovascular complications [2].

A range of mechanisms causing tissue damage and cardiovascular system dysfunction are triggered by diabetes mellitus. More specifically, the persistent hyperglycaemia acting on the various pathways underpinning enhanced oxidative stress has been identified as the most probable source of these mechanisms [3]. Diabetic cardiomyopathy and/or vascular dysfunction are in part the outcome of myocardial fibrosis and inflammation, which are therefore key determinants of cardiac dysfunction [4, 5]. Furthermore, cardiac ischemia and dysfunction are considered to be caused by alterations in oxidative stress and hyperglycaemia [6]. Alongside diabetes mellitus, several other disorders are accompanied by the cardiovascular risk of extension of heart-rate corrected QTc and QT interval duration that serves as markers of delayed cardiac repolarisation [7]. A number of studies have indicated that diabetics are more likely to develop cardiac conduction irregularities, such as high-degree atrioventricular (AV) block [8].

A non-toxic and cheap compound, zingerone (4-(4-hydroxy-3-methoxyphenyl)-2-butanone) is the *Zingiber officinale* ingredient with the lowest degree of pungency and has a range of pharmacological actions. Various experimental models have proposed that zingerone protects against tissue inflammatory damage, toxicity triggered by drugs, and oxidative stress caused by radiation [9]. Our team has shown that in vitro incubation with zingerone protects from exaggerated vasoconstriction in diabetic aortae. An effect seems to be mediated through a zingerone vasodilator effect through NO–and guanylate cyclase stimulation [10]. In the heart, zingerone protects rat heart against isoproterenol-induced myocardial infarction [11]. In addition, zingerone inhibits the hyperlipidaemia and cardiac hypertrophy in rats induced by isoproterenol [12].

In the present study, the response of cardiac conductivity in non-diabetic and diabetic rats to zingerone is investigated to determine how zingerone treatment affects cardiac repolarisation in a rat model with diabetes induced with streptozotocin. Furthermore, this investigation is also concerned with the impact of zingerone on the oxidative stress markers 8-isoprostane and uric acid, the cardiac oxidation mediator Angiotensin AT_1_ receptor and the cardiac antioxidant enzyme catalase, as well as on anti-inflammatory (adiponectin) and pro-inflammatory cytokines (transforming growth factor beta1, TGFβ1).

## Materials and methods

### Chemicals

Sigma-Aldrich (St. Louis, MO, USA) provided the zingerone (vanillyacetone) and streptozotocin (STZ), while Troikaa Pharmaceuticals Ltd. (Gujarat, India) provided the ketamine and xylazine.

### Animals

Six-week-old male Wistar rats with a weight of 250g were obtained from King Abdul-Aziz University, KSA. They were kept in clear cages made of polypropylene and with good ventilation (3–4 rats in each cage), under constant environmental conditions of 22 ± 2°C temperature, 50–60% relative humidity and 12-hour day and night cycle. Unlimited rodent pellet food and purified water were provided to the rats. This study was carried out in accordance with the recommendations of the Implementing Regulations of the Law of Ethics of Research on Living Creatures in Saudi Arabia. The protocol was approved by the King Abdulaziz University Research Ethics Committee.

### Diabetes induction

The rats were subjected to overnight fasting before they were injected intraperitoneally (i.p.) with a fresh ice-shield solution of 50 mg/kg streptozocin (STZ, Sigma, St. Louis, MO, USA) that had been dissolved in cold distilled water prior to administration [13]. To deal with the hypoglycaemia caused by streptozocin, a 10% glucose solution was freely provided to the rats overnight. As explained in section 2.7, glucometry was used to assess the development of diabetes, with rats being considered diabetic if they exhibited levels of non-fasting blood glucose that exceeded 250 mg/dl. In the following seven weeks, these rats also developed cardiovascular complications [14].

### Zingerone administration

To make it more soluble, zingerone (vanillylacetone) was dissolved in distilled water with 0.1% ethanol and 1% Kolliphor (Sigma) and an intragastric tube was used for oral administration in a dosage of 20 mg/kg body weight, the same dosage employed by the majority of earlier studies studies [15–17].

### Research approach

Groups consisting of eight rats each were respectively administered each day the vehicle (control group, C), STZ and then zingerone vehicle (diabetic, D), STZ and then zingerone (diabetic treated with zingerone, DZ), or zingerone without prior administration of STZ (CZ). After the seven-week period of zingerone administration, the rats were subjected to anaesthesia with 100 mg/kg ketamine and 10 mg/kg xylazine. Subsequently, a surface electrocardiograph was used to record cardiac conductivity in keeping with established protocols [18]. The next step was collection of 1ml blood by making a small incision in the lower abdomen to access the vena cava as well as samples of heart. Prior to separation of serum into aliquots, the blood sample was left to undergo coagulation for half an hour at a temperature of 4°C and subjected to centrifugation at 3000 rpm and temperature of 4°C for 20 minutes. Saline was used to wash the heart samples. Each sample was sectioned in two, with one portion being snap frozen and placed into storage at -80°C to be analysed at a later date, and the other portion was fixed in neutral buffered formalin 1% solution for immunofluorescence analysis.

### Electrocardiographic recording

A Power Lab® system (AD Instruments, Bella Vista, Australia) linked to a PC executing LabChart professional software was used to record the ECG. The software was run with the ECG module to enable detection of the various ECG components [16]. QRS detection is based on methods described by Hamilton and Tompkins (Hamilton and Tompkins, 1986). The corrected formula (QTc_n_-B = QT/(RR/f)^1/2^, f = 150 ms) was used to calculate the heart rate-corrected QT duration (QTc_n_) in Wistar rats as described previously [19].

### Biochemical analysis

A glucometer (Accu-Chek®, Roche Diagnostics, Mannheim, Germany) was employed to conduct serial estimates of glucose in single drop blood samples following overnight fasting. Performance of this apparatus exceeds ISO 15197 [20]. Rat ELISA kits (Abcam, Cambridge, UK) were used according to the manufacturer’s guidelines to assess cardiac TGFβ1 and serum adiponectin. To collect urine, the rats were kept in separate metabolic cages for one day. Gravimetric analysis was conducted to measure the amount of urine collected, while urine samples were stored at -80°C for later assessment of 8-isoprostane using a sensitive rat enzyme immunoassay kit (R&D Systems, Inc., Minneapolis, MN, USA). was used to measure urinary 8-isoprostane.

### Measurement of lipid profile, catalase activity and body weight gain

Test kits (Crescent Diagnostics, Jeddah, KSA) were employed for spectrophotometric analysis to measure the levels of triglycerides (TG) and total cholesterol (TC). The measurement of low density lipoprotein (LDL) was done in keeping with [17]. Furthermore, kits (Bio Diagnostic, Cairo, Egypt) were used to determine catalase in cardiac tissue homogenate.

### Immunofluorescence studies

Established laboratory guidelines were followed in the performance of immunofluorescence staining of AT_1_ and TGFβ1 [21, 22]. The tissue was fixed in 10% neutral phosphate-buffered formalin solution, after which it was subjected to dehydration in increasing concentration of ethanol. The tissue was then cleared in xylene and fixed in paraffin. Cold methanol was used for perforation of 4 *μ*m tissue sections, after which citrate buffer was used to extract the antigen. A buffer consisting of goat serum and bovine serum albumin hindered non-specific binding. Mouse monoclonal anti-AT_1_ (1:133 dilution) and rabbit polyclonal anti- TGFβ1 (1:1000 dilution) were the primary antibodies employed for immunofluorescence staining, while Alexa Fluor 488 -conjugated goat anti-mouse and Alexa Fluor 594 -conjugated goat anti-rabbit (dilution, 1:200) were the secondary antibodies. A Zeiss Observer D1 microscope was used to visualise the slides and Zen software (Carl Zeiss, Gottingen, Germany) permitted the fluorescent images to be compared.

### Histopathological examination

Neutral formalin-fixed paraffin section (5μm) were stained by Harris’ hematoxylin-eosin and Masson’s trichrome stains, according to the standard procedures. The tissues were then examined by a blinded expert in order to detect any histopathological changes.

### Statistical analysis

Expression of values took the form of mean ± SEM. Statistical analysis consisted of analysis of variance (ANOVA) and Newman-Keuls’ post-hoc test (p<0.05 significance level) conducted with the Prism 5 software (Graph Pad, USA).

## Results

### Zingerone impact on blood glucose, weight gain and lipid profile

By comparison to the control rats, those that were injected with streptozotocin (50mg/kg) exhibited high levels of hyperglycaemia (p<0.05 significance level) that persisted until the end of the seven-week period. However, neither the hyperglycaemia in diabetic rats nor the level of blood glucose in control rats was markedly affected by daily administration of 20 mg/kg zingerone. Meanwhile, unlike the control rats, the rats with diabetes triggered by stretozotocin exhibited a significantly lower (p<0.05) body weight gain. Moreover, the decrease in body weight gain in the case of diabetic rats was significantly ameliorated (p<0.05) by zingerone administration, while in the case of control rats, the body weight gain was unaffected (Table 1).

**Table 1:** The impact of 20 mg/kg zingerone administered orally every day on blood glucose, body weight gain (BWG), and lipid profile, including total cholesterol (TC), triglycerides (TG), and low density lipoprotein (LDL-C) in rats with diabetes (D) induced through intraperitoneal injection with 50 mg/kg stretozotocin and in control rats (C).

Groups	Blood glucose level (mg/dl)	BWG %	TC (mg/dl)	TG (mg/dl)	LDL-C (mg/dl)
**C**	93.75±2.827	29.50.± 3.256	67.62±5.103	44.47±4.087	53.69±5.801
**D**	241.4*±38.03	5.588*±3.987	77.18±5.884	48.35±8.375	57.92±4.818
**DZ**	331.4±22.21	12.06^#^±3.151	71.76±3.104	45.11±7.169	58.17±4.688
**CZ**	95.86±2.988	26.32. ±2.046	71.87±3.966	35.79±5.467	54.75±2.981

Expression of data takes the form of mean ± standard error for n = 5–9 rats per group. According to the one-way ANOVA and Newman Keuls’ post-hoc test, by comparison to the control group value and diabetic rats value

*P<0.05 and

^#^P<0.05, respectively.

### Zingerone impact on cardiac ECG

**Fig 1:**
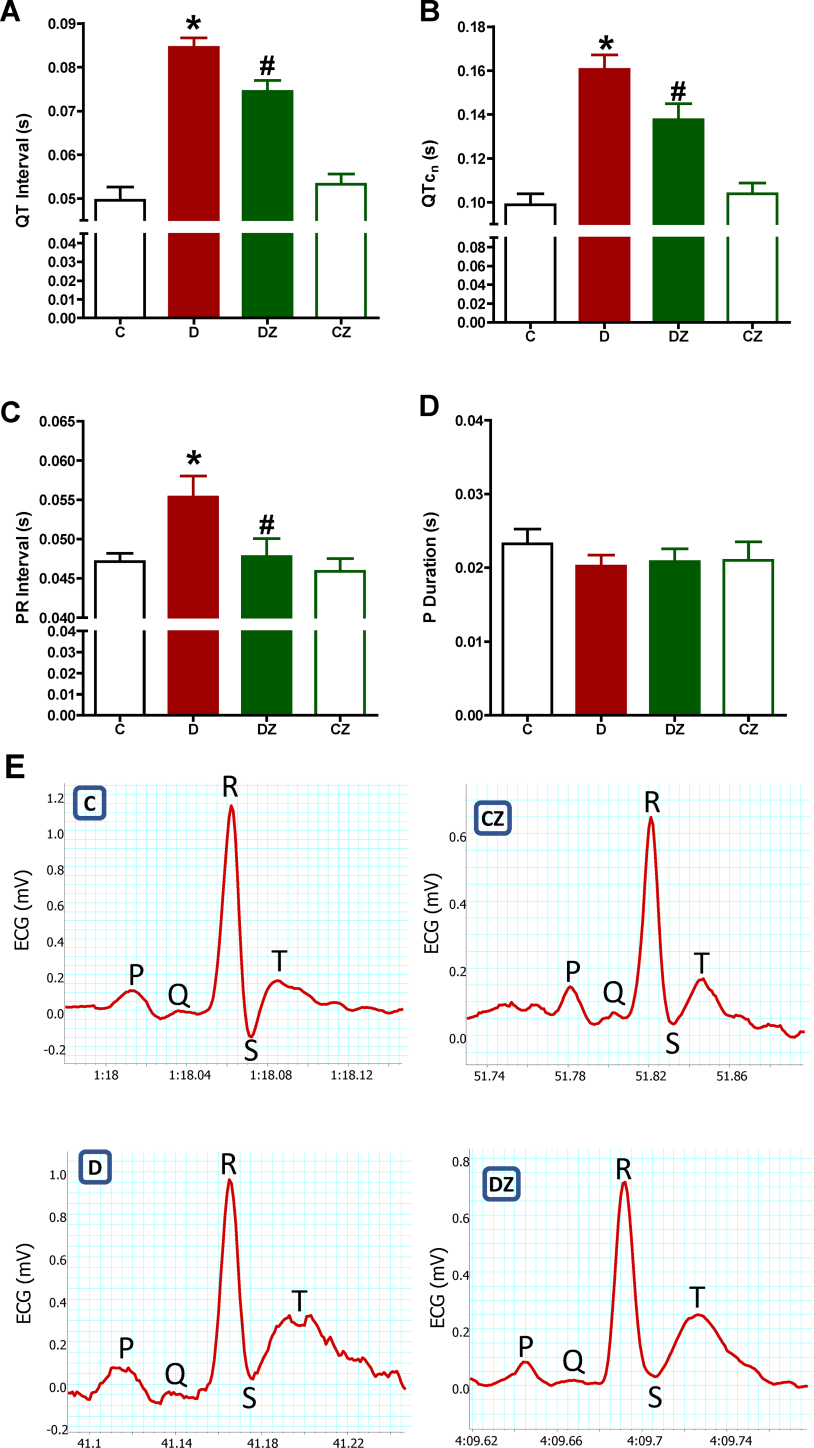
The impact of 20 mg/kg zingerone orally administered every day on cardiac ECG parameters QT (A), QTc (B), PR interval (C) and P-duration (D) in rats with diabetes (D) induced with 50 mg/kg streptozotocin and in control rats. (E) are representative cardiac ECG recordings. Expression of values takes the form of mean ± SD for N = 6–8 rats. According to the one-way ANOVA and Newman Keuls’ post-hoc test, by comparison to the control group value and D group value, *P<0.05 and ^#^P<0.05, respectively.

As indicated by the considerable expansion in QT duration and QTc duration interval by comparison to control (p<0.05), cardiac repolarisation was extended by streptozotocin-induced diabetes. However, by comparison to diabetic rats, those who were administered zingerone exhibited improved diabetes-related extended cardiac repolarisation, as reflected in the substantial reduction in QT and QTc durations (p<0.05). On the other hand, the QT and QTc durations in control rats that were administered zingerone was insignificantly affected, as can be seen in Fig 1A, 1B and 1E. The considerable (p<0.05) expansion in the PR duration suggested a correlation between diabetes and AV delay signs, whereas no effect on P-wave duration was observed by comparison to control. The PR duration was significantly reduced (p<0.05) when zingerone was administered to diabetic rats, suggesting that it ameliorated AV delay, but it did not influence P-wave duration. Furthermore, neither the PR duration nor the P-duration values were significantly affected in control rats that were administered zingerone (Fig 1C, 1D and 1E).

### Zingerone impact on fibrosis

**Fig 2:**
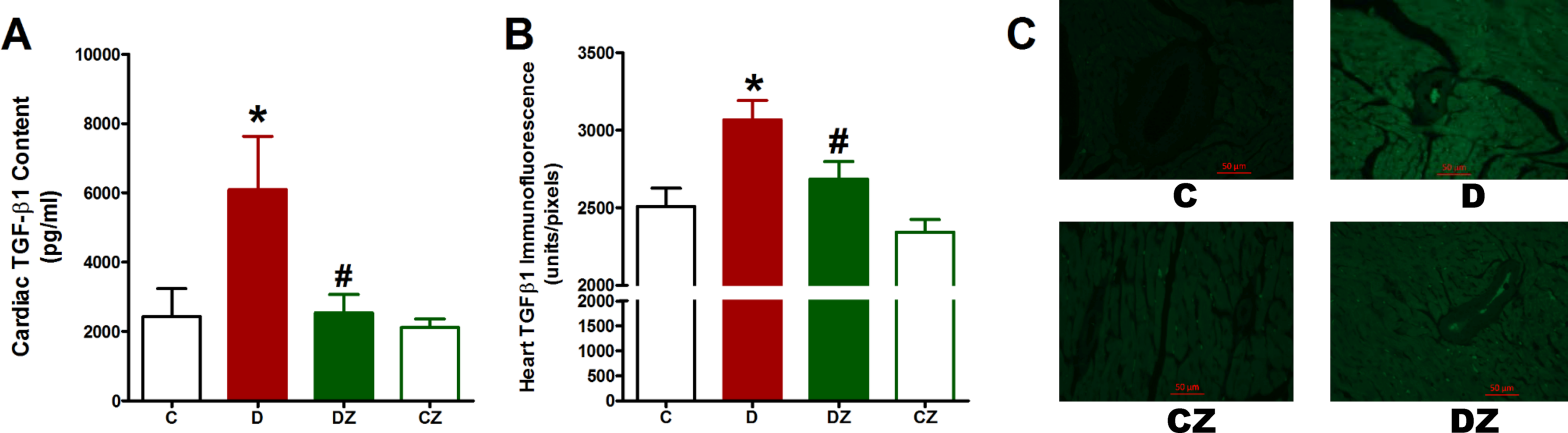
The impact of 20 mg/kg zingerone (Z) orally administered every day on cardiac tissue transforming growth factor β1 (TGFβ1) content (A) and immunofluorescence (B) in rats with diabetes (D) induced with 50 mg/kg streptozotocin and in control rats. Expression of values takes the form of mean ± SD for N = 6–8 rats. According to the one-way ANOVA and Newman Keuls’ post-hoc test, by comparison to the control group value and D group value, *P<0.05 and ^#^P<0.05, respectively. The right-hand side micrographs (C) are representative fluorescence images showing immunofluorescence of 5μm thick heart cross-sections stained with TGFβ1 antibodies and then with Alexa fluor conjugated secondary antibodies.

The cardiac content of the fibrogenic cytokine TGFβ1 was significantly increased in streptozotocin-induced diabetic rats (p<0.05) compared to control rats, suggesting a correlation between the diabetic cardiac arrhythmias and fibrosis signalling activation (Fig 2). Meanwhile, TGFβ1 levels was decreased by zingerone (p<0.05), showing the potential of this compound in ameliorating diabetes-related fibrosis signalling activation (Fig 2). Likewise, cardiac TGFβ1 immunofluorescence was significantly higher (p<0.05) in diabetic rats, but zingerone administration substantially decreased it. On the other hand, cardiac TGFβ1 immunofluorescence in control rats was unaffected by zingerone (Fig 2).

**Fig 3:**
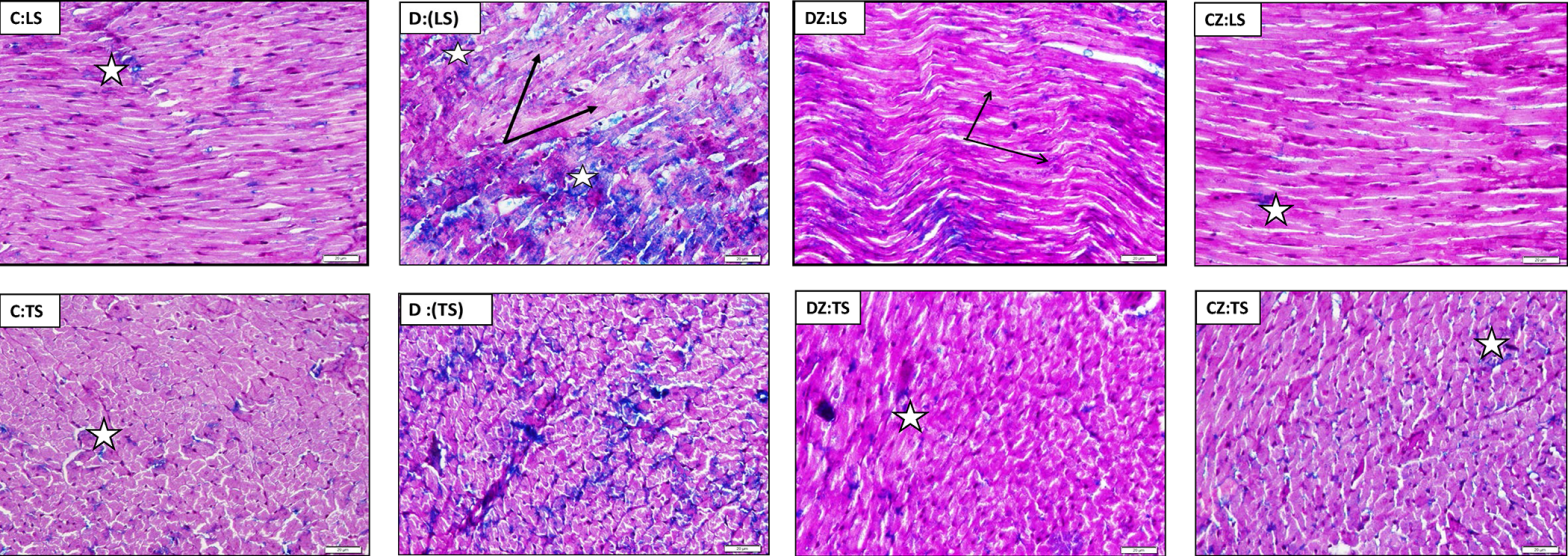
The impact of 20 mg/kg zingerone (Z) orally administered every day on collagen deposition in heart longitudinal (LS) and transverse (TS) sections stained with Masson Trichrome of rats with diabetes (D) induced with 50 mg/kg streptozotocin and in control (C) rats. Collagen fibres are stained blue. The solid arrows indicate degenerated cardiac muscle fibers, hollow stars indicate patches of fibrous tissue.

In confirmation of TGFβ1 results, Masson trichrome stained cardiac sections showed marked increase in collagen fibers at the site and between the degenerated cardiac muscle fibers in diabetic rats, while zingerone administration alleviated both collagen deposition and muscle degeneration (Fig 3).

### Zingerone impact on inflammation

**Fig 4:**
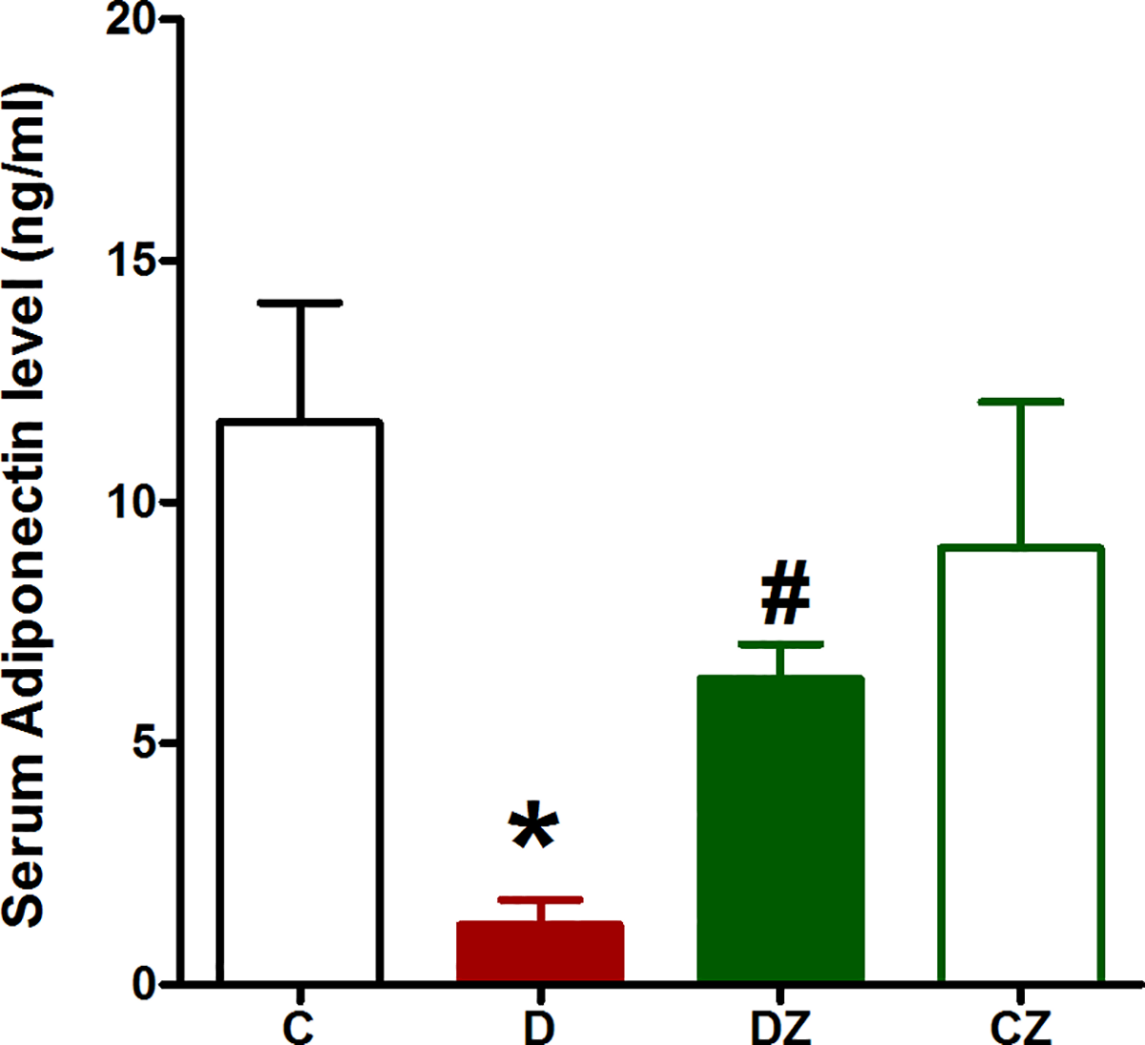
The impact of 20 mg/kg zingerone (Z) orally administered every day on serum level of adiponectin in rats with diabetes (D) induced with 50 mg/kg streptozotocin and in control rats. Expression of values takes the form of mean ± SD for N = 6–8 rats. According to the one-way ANOVA and Newman Keuls’ post-hoc test, by comparison to the control group value and D group value, *P<0.05 and ^#^P<0.05, respectively.

The levels of anti-inflammatory cytokine adiponectin were significantly reduced in the serum of streptozotocin-induced diabetic rats (p<0.05) compared to control rats, suggesting a correlation between diabetes and low-grade inflammation (Fig 4). Meanwhile, adiponectin levels were increased by zingerone (p<0.05), showing the potential of this compound in ameliorating diabetes-related low-grade inflammation (Fig 4).

### Zingerone impact on oxidative stress

**Fig 5:**
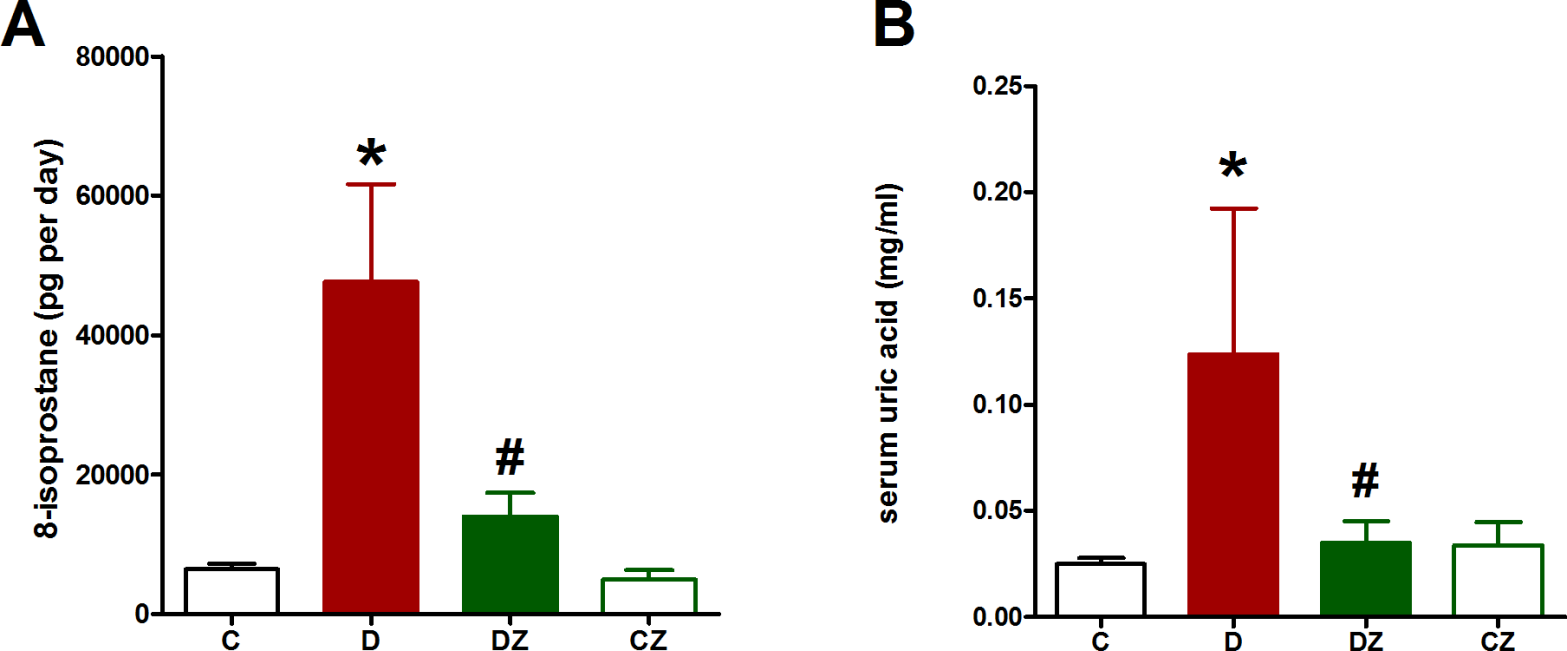
The impact of 20 mg/kg zingerone orally administered every day on urine 8-isoprostane level and serum uric acid level in rats with diabetes (D) induced with 50 mg/kg streptozotocin and in control rats. Expression of values takes the form of mean ± SD for N = 6–8 rats. According to the one-way ANOVA and Newman Keuls’ post-hoc test, by comparison to the control group value and D group value, *P<0.05 and ^#^P<0.05, respectively.

Fig 5 shows that, by comparison to control, the urine 8-isoprostane and serum uric acid levels were significantly (p<0.05) high in rats with diabetes induced by streptozotocin, suggesting excessive levels of oxidative stress. Conversely, urine 8-isoprostane and serum uric acid levels displayed a significant reduction (p<0.05) after zingerone was administered, indicating a decrease in oxidative stress (Fig 5).

**Fig 6:**
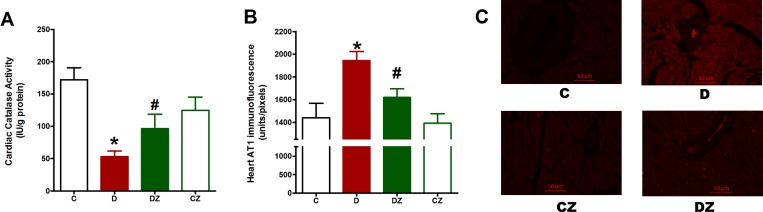
The impact of 20 mg/kg zingerone orally administered every day on cardiac catalase activity (A) angiotensin receptor type 1 (AT_1_) immunofluorescence (B) in rats with diabetes (D) induced with 50 mg/kg streptozotocin and in control rats. Expression of values takes the form of mean ± SD for N = 6–8 rats. According to the one-way ANOVA and Newman Keuls’ post-hoc test, by comparison to the control group value and D group value, *P<0.05 and ^#^P<0.05, respectively. The right-hand side micrographs (C) are representative fluorescence images showing immunofluorescence of 5μm thick heart cross-sections stained with AT_1_ antibodies and then with Alexa fluor conjugated secondary antibodies.

Inside the diabetic heart, catalase activity was suppressed while immunofluorescence for angiotensin receptor AT_1_ was significantly high (p<0.05), suggesting suppression of antioxidant enzymes and activation angiotensin system activation in diabetes (Fig 6). This streptozotocin-induced inhibition in catalase activity and rise in AT_1_ immunofluorescence in heart tissue from rats with diabetes was considerably reduced by zingerone administration (Fig 6).

## Discussion

The protective effect of the ginger component zingerone against diabetes-related delayed ventricular repolarisation and AV conduction has never been investigated until this study. In rats with diabetes, zingerone effectively hindered cardiac delayed repolarisation and AV conduction from occurring, whilst having no impact on the developed hyperglycaemia. Several results have been obtained that validate the ability of zingerone to prevent diabetes-related cardiac arrhythmias and inflammation: (i) zingerone ameliorated the extended cardiac repolarisation in diabetic rats; (ii) the diabetes-related delayed AV conduction was suppressed by zingerone; (iii) zingerone determined a decrease in the levels of urine 8-isoprostane, serum uric acid and cardiac AT_1_, while it increased cardiac catalase activity, indicating its capability to reduce diabetes-related oxidative stress; and (iv) the levels of cardiac TGFβ1 and serum adiponectin were decreased, reflecting the potential of zingerone to alleviate diabetes-related low-grade inflammation. It can be concluded based on these findings that zingerone inhibits oxidative stress and low-grade inflammation and thus protects against diabetes-related cardiac delayed repolarisation and AV conduction.

A number of diseases, including diabetes-related cardiac dysfunction, are related to the major cardiovascular risk factor of delayed cardiac repolarisation [23]. By comparison to control rats, the rats with diabetes in this study demonstrated considerable elevation in QT and QTc, which was indicative of delayed ventricular repolarisation. It is not yet fully understood how prolonged repolarisation is involved in type II diabetes, even though repolarisation heterogeneity is noted [24]. This study has demonstrated that zingerone ameliorated delayed repolarisation, involving ROS derived from xanthine oxidase. Although it is still uncertain what the molecular mechanism underpinning diabetes-related delayed repolarisation is, it appears that it consists of suppression of voltage gated K^+^ channels, ROS-based oxidation and p90 activation [25] and/or cardiac sodium channels and protein kinase II dependent on calcium/calmodulin [26].

Compared to non-diabetic individuals, those with diabetes seem to be more likely to experience atrioventricular (AV) delay or block [27]. Earlier studies have provided proof of the close association between AV delay and diabetes [8, 28]. Similarly, this study observed a correlation between diabetes and AV delay, but administration of zingerone counteracted it.

Cardiac fibrosis plays a very important role in promoting cardiac arrhythmias as fibrosis disrupts the normal electrical connectivity of cardiac tissue [29]. ELISA and immunofluorescence measurements in the current study prove that zingerone significantly ameliorated the fibrosis signalling in diabetic hearts as indicated by suppressing the inflammatory fibrogenic cytokine, transforming growth factor β1 (TGFβ1). TGF-*β* 1 improves extracellular matrix synthesis and thus serves as an important fibrogenic cytokine in a number of tissues [30]. In addition, in the current study, the marked cardiac muscle fibrosis and degeneration observed in diabetic sections were alleviated by zingerone treatment. Fibrosis signalling is closely associated with the inflammatory/oxidative status. Meanwhile, excessive cardiac NADPH oxidase activity in rats with high blood pressure was reported to be suppressed by TGFβ1 signalling inhibition [31]. A causal role of oxidative stress in muscular dystrophies pathogenesis [32] and glycogen deposition disorders [33] has been documented.

The onset and development of arrhythmogenic cardiomyopathy is dependent on low-grade inflammation [34, 35]. Therefore, this study investigated whether the protective effect exhibited by zingerone against diabetes-related cardiac diastolic dysfunction was due to a disruption in the balance of pro-inflammatory and anti-inflammatory mechanisms. In relation to this, the results of the ELISA measurements indicated that the levels of the anti-inflammatory cytokine adiponectine in the serum of diabetic rats were maintained by zingerone. Counteracting cellular inflammation and oxidative stress is the function performed by adiponectin [36]. One study reported that excessive expression of adiponectin afforded transgenic mice tolerance to left ventricular hypertrophy and diastolic dysfunction caused by aldosterone [37]. The positive impact of zingerone on both cardiac pro-inflammatory and anti-inflammatory cytokines that was observed in this study is consistent with the observations of earlier studies on this compound. One study indicated that the upregulation of myocardial pro-inflammatory genes (TNF-alpha, IL-1beta and IL-6) and coronary thrombosis in rats with myocardial infarct induced by isoproterenol were both stopped by zingerone pre-treatment [38]. Furthermore, another study on CC14-intoxicated rats reported an association between the hepatoprotective effect of zingerone and its inhibiting effect on inflammation via suppression of activation of upstream mitogen-induced protein protein kinases [39].

In addition, there can be no doubt that oxidative stress plays an important role in the onset of diabetes and related protein kinases [39]. Inactivation and dysfunction are caused by the interaction between the many different cellular proteins and the reactive oxygen species [24] produced by diabetes-related advanced glycation end-products and enzymes [40]. Relaxation of the cardiovascular system is impeded by ROS through absorption of secreted NO, unbinding of endothelial NO synthase and activation of inflammatory gene transcription [41]. Previous studies have reported that diabetic animals exhibited increased aortic ROS [42] and nuclear factor kappa B [13], and these elevated levels were closely associated with dysfunctional NO synthesis and endothelial-based relaxation [43]. This study obtained elevated levels of urine 8-isoprostane and serum uric acid (markers of central oxidative stress) in the animal models with diabetes, but these levels were reduced when zingerone was administered. Furthermore, zingerone hindered the excessive expression of the AT_1_ receptor (a major oxidative stress signaling pathway), while it improved catalase activity (an important antioxidant enzyme) in the cardiac tissue of diabetic rats. An earlier study also reported that zingerone displayed antioxidant effect associated with its radical scavenging activity [44]. Moreover, zingerone showed significant antioxidant activities that mediated its cardio-protection [11]. The zingerone antioxidant activities seems to be through preventing cardiac mitochondrial oxidative stress, Ca^2+^ overload and ATP depletion in isoproterenol-induced myocardial infarcted rats [45].

Since the developed hyperglycaemia of diabetic rats was unaffected by zingerone, it appears that the protective effect displayed by this compound against delayed cardiac repolarisation is not a result of alleviating diabetes but it is rather a form of direct cardiovascular protection. Nevertheless, this observed effect was not significantly potent.

## Conclusions

To summarise, the findings obtained by this study confirm that diabetes-related delayed cardiac repolarisation and AV conduction are ameliorated by orally administered zingerone, as well as that the inhibition of cardiac fibrosis and associated inflammation-oxidative stress by zingerone underpins its protective effect.

## Supporting information

S1 Appendix Supporting information file. (PZF)

## Abbreviations

AV: atrioventricular
AT_1_: Angiotensin receptor type 1
C: control group
CZ: zingerone without prior administration of STZ
D: diabetic group
DZ: diabetic treated with zingerone
LDL: low density lipoprotein
ROS: reactive oxygen species
TC: total cholesterol
TGFβ1: transforming growth factor beta1
STZ: Streptozotocin
TG: triglycerides
